# Impact of high rheumatoid factor levels on treatment outcomes with certolizumab pegol and adalimumab in patients with rheumatoid arthritis

**DOI:** 10.1093/rheumatology/keae435

**Published:** 2024-09-02

**Authors:** Josef S Smolen, Peter C Taylor, Yoshiya Tanaka, Tsutomu Takeuchi, Motomu Hashimoto, Carlos Cara, Bernard Lauwerys, Nicola Tilt, Baran Ufuktepe, Ricardo M Xavier, Alejandro Balsa, Jeffrey R Curtis, Ted R Mikuls, Michael Weinblatt

**Affiliations:** Division of Rheumatology, Department of Medicine, Medical University of Vienna, Vienna, Austria; Nuffield Department of Orthopaedics, Rheumatology and Musculoskeletal Sciences, Botnar Research Centre, University of Oxford, Oxford, UK; The First Department of Internal Medicine, University of Occupational and Environmental Health, Kitakyushu, Japan; Department of Rheumatology and Applied Immunology, Saitama Medical University, Saitama, Japan; Division of Rheumatology, Department of Internal Medicine, School of Medicine, Keio University School of Medicine, Tokyo, Japan; Department of Clinical Immunology, Osaka Metropolitan University, Osaka, Japan; UCB Pharma, Madrid, Spain; UCB Pharma, Brussels, Belgium; UCB Pharma, Slough, UK; UCB Pharma, Istanbul, Turkey; Faculdade de Medicina, Universidade Federal do Rio Grande do Sul, Hospital de Clínicas de Porto Alegre, Brazil; Rheumatology Unit, La Paz University Hospital, Madrid, Spain and Institute for Health Research (IdiPAZ), Madrid, Spain; Division of Clinical Immunology and Rheumatology, University of Alabama, Birmingham, AL, USA; Division of Rheumatology and Immunology, University of Nebraska Medical Center and VA Nebraska-Western Iowa Health Care System, Omaha, NE, USA; Division of Rheumatology, Inflammation and Immunity, Brigham and Women’s Hospital, Harvard Medical School, Boston, MA, USA

**Keywords:** RA, RF, TNF inhibitor

## Abstract

**Objectives:**

To assess the impact of baseline RF level on drug concentrations and efficacy of certolizumab pegol [CZP; TNF inhibitor (TNFi) without a crystallizable fragment (Fc)] and adalimumab (ADA; Fc-containing TNFi) in patients with RA.

**Methods:**

The phase 4 EXXELERATE study (NCT01500278) was a 104-week, randomized, single-blind (double-blind until week 12; investigator-blind thereafter), head-to-head study of CZP *vs* ADA in patients with RA. In this *post hoc* analysis, we report drug concentration and efficacy outcomes stratified by baseline RF quartile (≤Q3 or >Q3).

**Results:**

Baseline data by RF quartiles were available for 453 CZP-randomized and 454 ADA-randomized patients (≤Q3: ≤204 IU/ml; >Q3: >204 IU/ml). From week 12, the area under the curve (AUC) of ADA concentration was lower in patients with RF >204 IU/ml *vs* patients with RF ≤204 IU/ml; the AUC of CZP concentration was similar in patients with RF ≤204 IU/ml and >204 IU/ml. For patients with RF ≤204 IU/ml, disease activity score (DAS28)-CRP was similar between CZP- and ADA-treated patients through week 104. For patients with RF >204 IU/ml, mean DAS28-CRP was lower in CZP- *vs* ADA-treated patients at week 104. The proportion of patients with RF >204 IU/ml achieving DAS28-CRP low disease activity at week 104 was greater in CZP- *vs* ADA-treated patients.

**Conclusion:**

CZP was associated with maintained drug concentration and efficacy in patients with RA and high RF and may therefore be a more suitable therapeutic option than TNFis with an Fc fragment in these patients.

**Trial registration:**

Clinicaltrials.gov, http://clinicaltrials.gov, NCT01500278

Rheumatology key messages

- Patients with RA and high RF have poorer responses to TNF inhibitors with an Fc.
- Here, CZP (Fc-free) had consistent efficacy and plasma concentration, regardless of baseline RF.
- CZP may be more suitable than TNFis with an Fc for patients with RA and high RF.

## Introduction

RF is an autoantibody directed against the crystallizable fragment (Fc) region of IgG, and is characteristically found in patients with RA [1, 2]. RF itself can be present in multiple Ig isotypes (IgM, IgA and IgG) but, in clinical practice, IgM RF is the most commonly measured isotype; around 60–80% of patients with RA have detectable levels of IgM RF [3, 4]. In patients with RA, high RF levels are considered a poor prognostic factor and are associated with more aggressive and destructive disease, higher disease activity, higher cardiovascular risk and higher risk of radiographic progression [5–8].

TNF inhibitors (TNFis), including certolizumab pegol (CZP), adalimumab (ADA), etanercept (ETA), golimumab (GOL) and infliximab (IFX) are an effective treatment option for many patients with RA [9–18]. TNFis appear to have similar efficacy in large cohorts or trials in RA, such as the EXXELERATE study [19]. Despite all TNFis having the same mechanistic target, they bear different structural features. For example, CZP is a TNFi without an Fc fragment whereas other TNFis possess an Fc fragment.

Of note, a decreased response to treatment with TNFis in patients with RA has been associated with RF positivity, particularly with high levels of RF [20–26]. More recent preliminary data suggest that this decreased response may be associated with the presence of an Fc fragment [27]. Patients with RA and higher RF levels may therefore have a better response to therapy with a TNFi without an Fc fragment compared with those with an Fc fragment [28], though further studies are needed to support this observation. Indeed, in a *post hoc* analysis of six pooled trials, CZP showed similar efficacy across baseline RF level quartiles in patients with early and established RA [27]. In another retrospective analysis of patients with RA, serum drug levels of IFX and ADA (Fc-containing TNFis) at six months were significantly lower in patients who had higher RF levels at baseline compared with those with lower RF levels, while CZP levels remained consistent across baseline RF levels [29]. IFX- and ADA-treated patients with high baseline RF levels dropped out more frequently with secondary non-response than those receiving CZP (80% *vs* 75% *vs* 33%, respectively; *P* = 0.002) [29]. These findings suggest that IFX and ADA may be bound by RF, leading to accelerated drug clearance and consequently low drug levels and poorer clinical outcomes. However, these data were obtained using indirect comparative analyses rather than assessment of head-to-head studies.

We hypothesized that high RF levels may result in enhanced drug clearance and reduced efficacy of TNFis with an Fc fragment compared with those without an Fc fragment (such as CZP). In light of this, we assessed the efficacy and drug concentrations of CZP and ADA in patients with RA stratified by RF level at baseline in a *post hoc* analysis of the EXXELERATE study (a head-to-head trial which compared CZP to ADA).

## Patients and methods

### Study design and patients

The phase 4 EXXELERATE study (NCT01500278) was a 104-week, randomized, single-blind (double-blind until week 12 and investigator blind thereafter), parallel-group, head-to-head, superiority trial of CZP *vs* ADA in patients with RA [19]. Eligible patients were aged 18 or over with a diagnosis of active RA, defined as Disease Activity Score 28-erythrocyte sedimentation rate (DAS28-ESR) >3.2, ≥4/28 swollen joints, and increased acute phase reactants [high sensitivity C-reactive protein (hsCRP) ≥10 mg/l and/or ESR ≥28 mm/h] at screening and baseline despite a minimum of 12 weeks of MTX treatment prior to screening. Patients were biologic DMARD (b-DMARD)-naïve and had prognostic factors for severe progression (positive RF and/or ACPA result) with at least 28 days of stable MTX treatment.

At baseline, patients were randomized 1:1 to CZP [400 mg at weeks 0, 2 and 4, then 200 mg every 2 weeks (Q2W)] and +MTX or ADA 40 mg Q2W + MTX. During the first 12 weeks of the study, patients receiving ADA were also administered placebo injections at weeks 0, 2 and 4 to maintain blinding during the administration of the loading dose of CZP. At week 12, patients were classified as responders [by achieving low disease activity (LDA): DAS28-ESR ≤3.2 or DAS28-ESR reduction ≥1.2 from baseline] or as non-responders. Patients classified as non-responders to the TNFi to which they were randomized were switched to the other TNFi with no washout period. Patients who switched to ADA received 40 mg ADA Q2W + MTX; patients who were switched to CZP received a loading dose of 400 mg CZP at weeks 12, 14 and 16, followed by CZP 200 mg Q2W + MTX. RF levels were measured by Roche Tina-quant^®^.

### Ethics approval

The EXXELERATE study protocol, amendments and patient informed consent were reviewed by a national, regional or Independent Ethics Committee (IEC) or Institutional Review Board (IRB). EXXELERATE was conducted in accordance with the current version of the applicable regulatory and International Conference on Harmonisation (ICH)-Good Clinical Practice (GCP) requirements, the ethical principles that have their origin in the principles of the Declaration of Helsinki, and the local laws of the countries involved. Full details of the ethics committee involved are available upon request. All patients provided written informed consent to participate in the study.

As this was a *post hoc* analysis of anonymized data, no ethics committee or institutional review board approvals were required. All the results presented in this article are in aggregate form, and no personally identifiable information was used for this study. All such approvals were obtained in the original trial [19].

### Outcomes

We report the following outcomes in CZP- and ADA-treated patients to week 104, stratified by baseline RF quartile across all patients (≤Q3 or >Q3; quartile cut-offs chosen to allow for a reasonable number of patients in each subgroup): drug plasma concentration (Sanquin test; performed centrally), mean DAS28-CRP and the proportion of patients achieving DAS28-CRP low disease activity (LDA); DAS28-CRP LDA number needed to treat (NNT); mean DAS28-ESR and the proportion of patients achieving DAS28-ESR LDA; clinical disease activity index (CDAI) score; simplified disease activity index (SDAI) score; the proportion of patients achieving CDAI LDA (CDAI ≤10) and SDAI LDA (SDAI ≤11); HAQ-disability index (HAQ-DI) score, and the proportion of patients achieving revised Boolean remission [30].

To assess whether the impact of high RF levels was mediated by anti-drug antibodies, a sensitivity analysis was performed after exclusion of patients with high levels of anti-drug antibodies (>50th percentile; threshold chosen arbitrarily, based on the knowledge that higher titers are associated with a higher prevalence of neutralizing antibodies; measured with Sanquin assays) for DAS28-CRP and DAS28-ESR, and the proportion of patients achieving DAS28-CRP LDA and DAS28-ESR LDA.

As a sensitivity analysis to assess whether outcomes are confounded by factors such as immune activity and to ensure our observations are specific to RF level, we also report DAS28-CRP and DAS28-ESR in CZP- and ADA-treated patients to week 104, stratified by ACPA quartile (≤Q3 or >Q3; measured with immunoassay, performed centrally).

### Analyses

Analysis for outcomes from weeks 0 to 12 included all patients with baseline and post-baseline efficacy measurements (full analysis set); analysis for outcomes beyond week 12 included randomized patients who received at least one dose of study drug after week 12 and had valid baseline, week 12 and post-week 12 efficacy measurements (week 12 full analysis set). At week 12, patients who were non-responders to the TNFi were randomized to at baseline switched to the other TNFi; outcomes were analyzed according to the treatment patients were on at the time of measurement.

The area under the curve (AUC) of CZP and ADA plasma concentrations were calculated from week 12 onwards and also did not include patients who switched treatment at week 12, so that results were not distorted by the loading dose of CZP.

Data were reported as observed case (OC) for all outcomes other than revised Boolean remission [non-responder imputation (NRI)]. DAS28-CRP LDA and DAS28-ESR LDA are reported as OC and NRI. Reported *P* values were not pre-specified and are therefore nominal, and so should be interpreted with caution.

## Results

### Patient disposition and baseline characteristics

Baseline data by RF quartiles were available for 453 CZP-randomized patients (≤Q3 [RF ≤204 IU/ml]: *n* = 334; >Q3 [RF >204 IU/ml]: *n* = 119) and 454 ADA-randomized patients (≤204 IU/ml: *n* = 347; >204 IU/ml *n* = 107). At week 12, 66 CZP-randomized patients switched to ADA and 59 ADA-randomized patients switched to CZP. Baseline demographics and characteristics were similar between ADA- and CZP-randomized groups with RF ≤204 IU/ml and >204 IU/ml, though patients with high RF levels had greater mean disease duration (Table 1). Smoking status at baseline was not available.

**Table 1.** Baseline demographics and patient characteristics stratified by RF level at baseline

	≤Q3 (RF ≤204 IU/ml)		>Q3 (RF >204 IU/ml)	
	CZP + MTX (*N* = 334)	ADA + MTX (*N* = 347)	CZP + MTX (*N* = 119)	ADA + MTX (*N* = 107)
Age, mean (s.d.), years	52.7 (12.4)	52.5 (12.9)	55.5 (11.7)	54.2 (12.3)
BMI (kg/m^2^), mean (s.d.)	28.37 (6.35)	28.09 (6.02)	28.77 (6.29)	27.38 (7.04)
Sex (male, %)	19.2	20.5	26.9	22.4
Baseline steroid use (yes, %)	47.9	55.9	68.1	60.7
Previous TNFi use (yes, %)	0.6	0.3	0.0	0.9
Disease duration (years), mean (s.d.)	5.54 (6.20)	5.45 (6.50)	7.54 (8.58)	6.94 (7.84)
Baseline DAS28-CRP				
*n*	332	346	118	106
Mean (s.d.)	5.52 (0.87)	5.65 (0.85)	5.89 (0.91)	5.91 (0.96)
Baseline DAS28-ESR				
*n*	334	346	118	107
Mean (s.d.)	6.40 (0.86)	6.49 (0.82)	6.72 (0.90)	6.70 (0.93)
Baseline ACPA (U/ml)				
*n*	333	345	119	106
Mean (s.d.)	381.85 (757.61)	568.56 (921.99)	1034.81 (1487.80)	1099.61 (1786.50)
MTX dose (mg/week), mean (s.d.)	17.5 (3.6)	18.1 (3.9)	17.8 (4.3)	17.9 (4.0)

Full analysis set.

ADA: adalimumab; CZP: certolizumab pegol; DAS28: DAS-28 joint count; Q3: third quartile; TNFi: TNF inhibitor.

### Drug concentrations stratified by RF level

**Figure 1.**
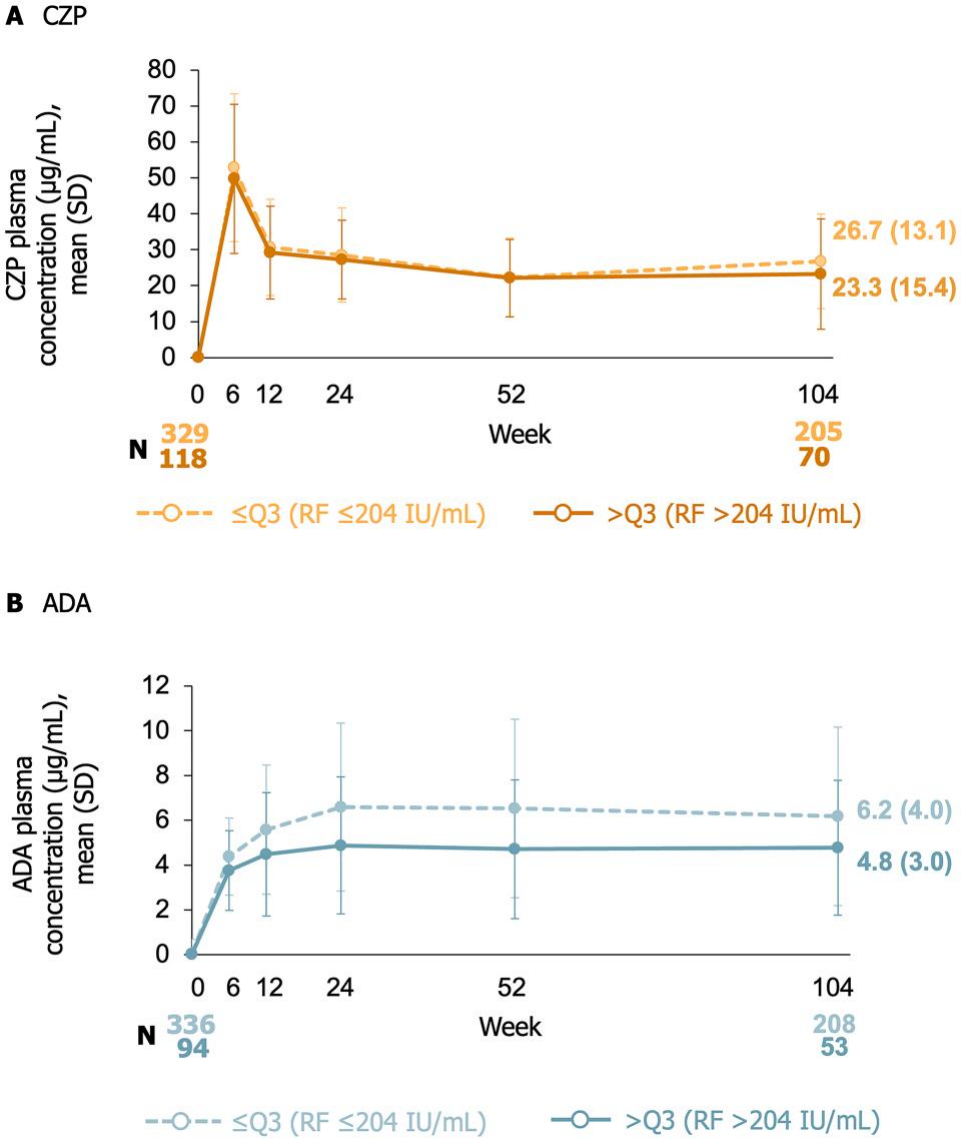
Mean drug plasma concentrations of (A) CZP and (B) ADA to week 104, stratified by RF quartile at baseline (OC). Full analysis set to week 12; week 12 full analysis set from week 18. Data reported according to the treatment patients were on at the time of measurement (i.e. any patients who had switched TNFi at week 12 were subsequently included in the arm for their new treatment, rather than the arm they were initially randomized to). ADA: adalimumab; CZP: certolizumab pegol; OC: observed case; Q3: third quartile; TNFi: TNF inhibitor

Figure 1 illustrates the drug plasma concentrations of CZP 200 mg and ADA 40 mg to week 104. From week 12 onwards, the AUC of ADA drug concentration was lower in patients with RF levels >204 IU/ml than in patients with RF levels ≤204 IU/ml; the AUC of CZP concentration was similar in patients with RF ≤204 IU/ml and >204 IU/ml.

### Efficacy stratified by RF level

**Figure 2.**
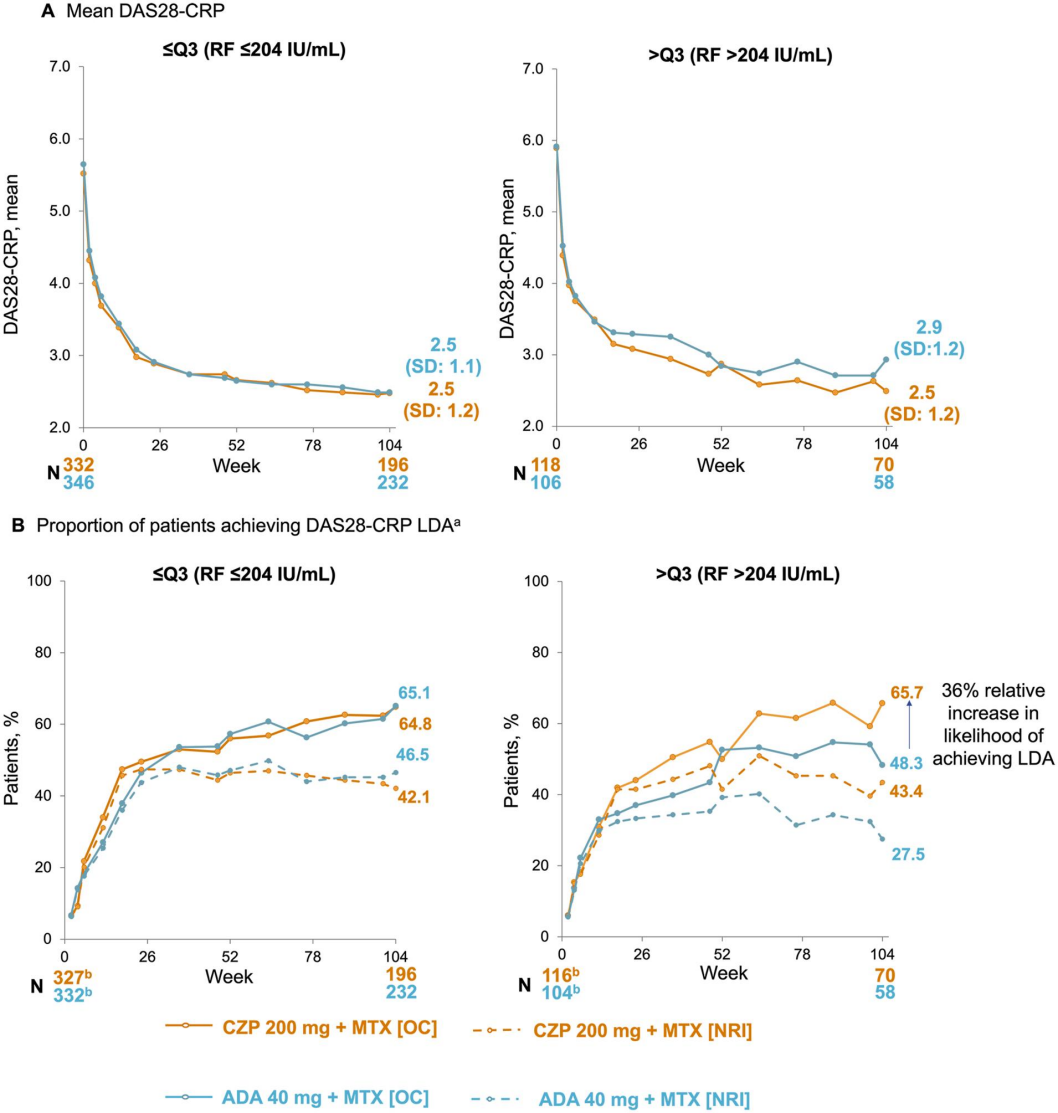
Response to CZP and ADA to week 104 measured by (A) DAS28-CRP and (B) DAS28-CRP LDA, stratified by RF quartile at baseline (OC; NRI). Full analysis set to week 12; week 12 full analysis set from week 18. Data reported according to the treatment patients were on at the time of measurement (i.e. any patients who had switched TNFi at week 12 were subsequently included in the arm for their new treatment, rather than the arm they were initially randomized to). *N* is for OC data. (a) Defined as DAS28-CRP ≤2.7. (b) *N* at week 2. ADA: adalimumab; CZP: certolizumab pegol; DAS28: Disease Activity Score-28 joint count; LDA: low disease activity; NRI: non-responder imputation; OC: observed case; Q3: third quartile; TNFi: TNF inhibitor

Clinical outcomes were poorer in patients with RF levels >204 IU/ml who were treated with ADA compared with those treated with CZP. For patients with RF ≤204 IU/ml, mean (s.d.) DAS28-CRP was similar between CZP- and ADA-treated patients at week 104 (CZP: 2.5 [1.2]; ADA: 2.5 [1.1]; nominal *P* = 0.917; Fig. 2). However, for patients with RF >204 IU/ml, mean (s.d.) DAS28-CRP was lower in CZP- *vs* ADA-treated patients at week 104 (CZP: 2.5 [1.2]; ADA: 2.9 [1.2]; nominal *P* = 0.046; Fig. 2). The proportion of patients with RF ≤204 IU/ml achieving DAS28-CRP LDA was similar between CZP- (127/196; 64.8%) and ADA-treated (151/232; 65.1%) patients at week 104 (OC data; Fig. 2). However, for patients with RF >204 IU/ml, the proportion achieving DAS28-CRP LDA was higher (36% relative increase; OC data) at week 104 in CZP- (46/70; 65.7%) *vs* ADA- (28/58; 48.3%) treated patients. Findings were similar when NRI was utilized (Fig. 2). NNT at week 104 for DAS28-CRP LDA in patients with RF ≤204 IU/ml was 52.1 for CZP *vs* ADA (95% CI: 193.5–297.6) and 5.5 (95% CI: 0.3–10.7) in patients with RF >204 IU/ml. A similar pattern was observed for DAS28-ESR and DAS28-ESR LDA (Supplementary Fig. S1, available at *Rheumatology* online).

**Figure 3.**
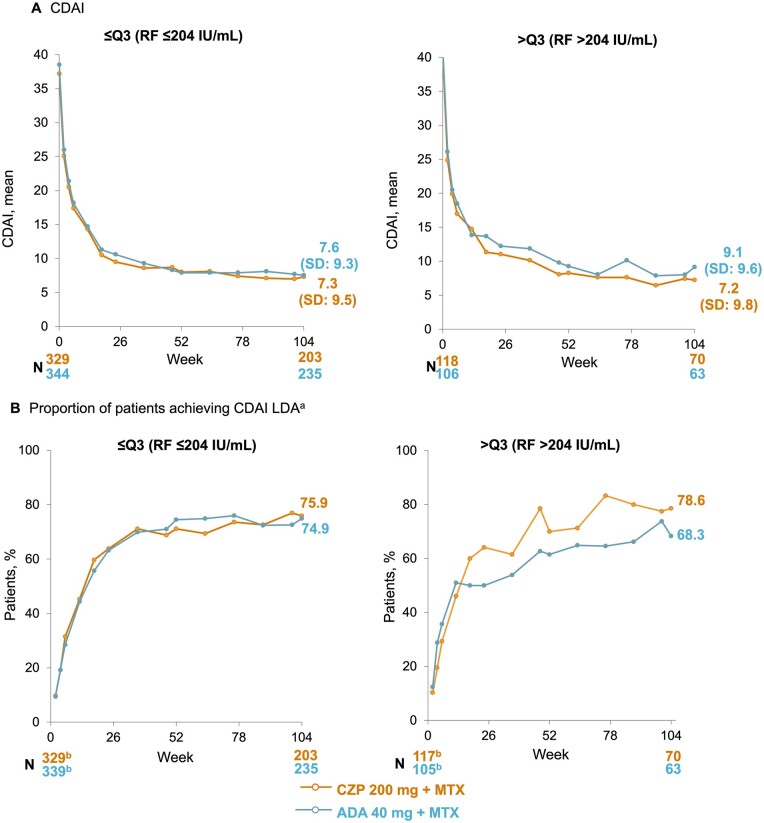
Response to CZP and ADA to week 104 measured by (A) CDAI and (B) CDAI LDA, stratified by RF quartile at baseline (OC). Full analysis set to week 12; week 12 full analysis set from week 18. Data reported according to the treatment patients were on at the time of measurement (i.e. any patients who had switched TNFi at week 12 were subsequently included in the arm for their new treatment, rather than the arm they were initially randomized to). (a) Defined as CDAI ≤10. (b) *N* at week 2. ADA: adalimumab; CDAI: Clinical Disease Activity Index; CZP: certolizumab pegol; OC: observed case; Q3: third quartile; TNFi: TNF inhibitor

Response to CZP and ADA, as measured by CDAI and CDAI LDA are illustrated in Fig. 3. SDAI and SDAI LDA are available in Supplementary Fig. S2, available at *Rheumatology* online. At week 104, mean (s.d.) CDAI was similar in CZP- and ADA-treated patients with RF levels ≤204 IU/ml (CZP: 7.3 [9.5]; ADA: 7.6 [9.3]). In patients with RF levels >204 IU/ml, mean (s.d.) CDAI was numerically lower in CZP- *vs* ADA-treated patients (CZP: 7.2 [9.8]; ADA: 9.1 [9.6]). The proportion of patients achieving CDAI LDA was similar for patients with RF levels ≤204 IU/ml at week 104 (CZP: 75.9%; ADA: 74.9%) while in patients with RF >204 IU/ml, the proportion of patients achieving CDAI LDA was higher in CZP- *vs* ADA- treated patients (CZP: 78.6%; ADA: 68.3%; Fig. 3). A similar pattern was observed for SDAI and SDAI LDA (Supplementary Fig. S2, available at *Rheumatology* online). HAQ-DI scores were similar between CZP- and ADA-treated patients with RF level ≤204 IU/ml and >204 IU/ml at week 104 (Supplementary Fig. S3, available at *Rheumatology* online).

Treatment response measured by revised Boolean remission is illustrated in Supplementary Fig. S4, available at *Rheumatology* online; from week 64, in patients with RF level >204 IU/ml, a higher proportion of CZP-treated patients achieved Boolean remission compared with ADA-treated patients (CZP: 8.5% at week 104; ADA: 2.9% at week 104).

### Sensitivity analyses: exclusion of patients with high levels of anti-drug antibodies

To assess whether the impact of high RF levels on treatment with ADA and CZP was mediated by anti-drug antibodies, DAS28-CRP, DAS28-ESR, DAS28-CRP LDA and DAS28-ESR LDA after exclusion of patients with high levels of anti-drug antibodies (>50th percentile) were analyzed. DAS28-CRP, DAS28-ESR, DAS28-CRP LDA and DAS28-ESR LDA after exclusion of patients with high levels of anti-drug antibodies (>50th percentile) are illustrated in Supplementary Figs S5 and S6, available at *Rheumatology* online, and are consistent with findings from the whole study population. For patients with RF levels ≤204 IU/ml, mean (s.d.) DAS28-CRP and DAS28-ESR were similar between CZP- and ADA-treated patients at week 104 (DAS28-CRP: 2.4 [1.1] CZP and 2.4 [1.1] ADA; DAS28-ESR: 2.9 [1.4] CZP and 3.0 [1.3] ADA) after exclusion of patients with high levels of anti-drug antibodies (Supplementary Fig. S5, available at *Rheumatology* online). However, for patients with RF levels >204 IU/ml, mean DAS28-CRP and DAS28-ESR were lower in CZP-treated patients compared with ADA-treated patients at week 104 (DAS28-CRP: 2.2 [0.8] CZP and 2.8 [1.1] ADA; DAS28-ESR: 2.9 [1.0] CZP and 3.5 [1.3] ADA) after exclusion of patients with high levels of anti-drug antibodies.

The proportions of patients achieving DAS28-CRP LDA and DAS28-ESR LDA after exclusion of patients with high levels of anti-drug antibodies were also similar in patients with RF levels ≤204 IU/ml at week 104 (67.8% CZP and 66.5% ADA; DAS28-ESR: 67.1% CZP and 62.1% ADA; Supplementary Fig. S6, available at *Rheumatology* online). There was differentiation, however, between patients with RF levels >204 IU/ml at week 104 (DAS28-CRP LDA: 78.0% CZP and 52.8% ADA; DAS28-ESR: 65.9% CZP and 44.4% ADA).

### Sensitivity analyses: efficacy stratified by ACPA level

**Figure 4.**
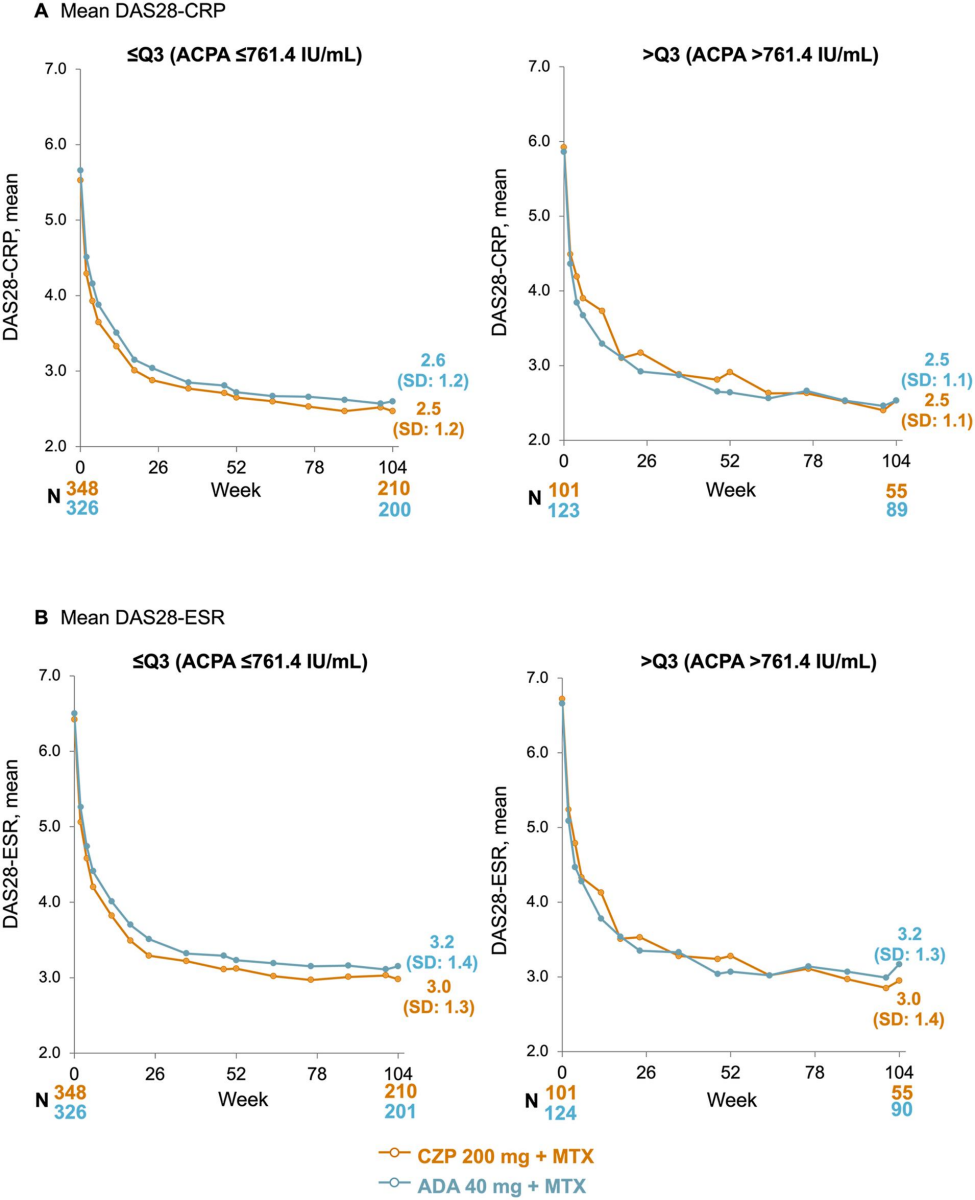
Response to CZP and ADA to week 104 measured by (A) DAS28-CRP and (B) DAS28-ESR, stratified by ACPA level at baseline (OC). Full analysis set to week 12; week 12 full analysis set from week 18. Data reported according to the treatment patients were on at the time of measurement (i.e. any patients who had switched TNFi at week 12 were subsequently included in the arm for their new treatment, rather than the arm they were initially randomized to). ACPA: anti-citrullinated protein antibody; ADA: adalimumab; CZP: certolizumab pegol; DAS28: Disease Activity Score-28 joint count; OC: observed case; Q3: third quartile; TNFi: TNF inhibitor

DAS28-CRP and DAS28-ESR were similar through Weeks 0–104 between CZP- and ADA-treated patients with both ACPA levels ≤761.4 IU/ml and >761.4 IU/ml (Fig. 4). At week 104, in patients with ACPA levels >761.4 IU/ml, mean (s.d.) DAS28-CRP was 2.5 (1.1) in CZP-treated patients and 2.5 (1.1) in ADA-treated patients; mean (s.d.) DAS28-ESR was 3.2 (1.3) in ADA-treated patients and 3.0 (1.4) in CZP-treated patients. No strong association between RF and ACPA levels at baseline was observed (Table 2).

**Table 2.** Numbers of patients with ACPA and RF levels in matching quartiles

ACPA level (U/ml)					
		≤Q1 (≤34.5)	>Q1 − ≤Q2 (>34.5 − ≤186.5)	>Q2 – ≤Q3 (>186.5 – ≤761.4)	>Q3 (>761.4)
RF level (IU/ml)	≤Q1 (≤32.0)	95	65	45	28
	>Q1 – ≤Q2 (>32.0 – ≤75.0)	62	56	55	48
	>Q2 – ≤Q3 (>75.0 – ≤204.0)	42	51	69	62
	>Q3 (>204.0)	27	54	57	87

Full analysis set. Five patients had missing values. Each value represents the number of patients with both RF and ACPA levels within the specified ranges at baseline.

Q: quartile.

## Discussion

This *post hoc* analysis of the EXXELERATE study provides a direct comparison of CZP *vs* ADA in patients with RA and high RF levels. In patients with RF levels in the highest quartile, clinical outcomes were more favourable in those who were treated with CZP compared with patients who received ADA. This was observed over a period of 104 weeks; in the original EXXELERATE trial, no difference in treatment response was observed between CZP- and ADA-treated patients at 12 weeks. Further, a study by Lacroix, 2009 demonstrated a loading dose effect of CZP lasting up to week 10–12, with peak CZP plasma concentration observed between weeks 4–6 [31]. These observations suggest that the favourable clinical outcomes reported here in CZP-treated patients with high RF levels were not due to the initial CZP loading dose [19]. Further, drug concentrations were lower in patients with high RF levels compared with lower RF levels in ADA-treated patients, while such a difference was not observed in CZP-treated patients. The results of sensitivity analyses indicate that these findings were not confounded by factors such as the presence of anti-drug antibodies or ACPA levels (immune activity). The lack of any strong association between RF and ACPA levels at baseline may further contextualize why the observations made in patients with high RF levels were not reproduced in patients with high ACPA levels.

In a previous analysis of the effect of ADA plasma concentration on treatment response among patients with high RF levels, higher ADA levels were associated with better response and improved drug survival [32]. In that study, a lower therapeutic threshold of 6.0 mg/l was suggested [32]; in this *post hoc* analysis, plasma ADA levels reached 4.8 μg/ml at week 104 in patients with RF >204 IU/ml, which indicates that the lower serum drug concentration in ADA-treated patients with high levels of RF may have led to the lower efficacy compared with CZP. On the other hand, CZP-treated patients with RA and high RF levels had similar drug concentrations and maintained clinical outcomes compared with those with lower RF levels.

In patients with RA and RF levels >204 IU/ml, the proportion of patients achieving DAS28-CRP LDA and DAS28-ESR LDA at week 104 were 36% and 38% higher in CZP-treated patients relative to ADA-treated patients, respectively; there was little difference between CZP- and ADA-treated patients with RF levels ≤204 IU/ml. The temporary dip in DAS28-CRP LDA at week 52 in CZP-treated patients with RF >Q3 can be explained by the intrinsic variability of data in clinical trials; the present findings demonstrate a similar degree of variation as was reported in the original EXXELERATE paper. The response to CZP and ADA, as measured by CDAI and SDAI, was also greater in patients with RA treated with CZP compared with ADA in patients with RF levels >204 IU/ml, but not in those with RF levels ≤204 IU/ml.

In this analysis, the differences in CDAI observed between CZP- and ADA-treated patients with high RF levels were clinically important. The minimal clinically important difference cut-off for CDAI has previously been defined as 1 when CDAI is <10 [33]. Here, in patients with RF levels >204 IU/ml, CDAI was 1.9 points lower in CZP-treated patients compared with ADA-treated patients at week 104. Furthermore, NNT at week 104 for DAS28-CRP LDA was ∼10 times greater in patients with RF levels ≤204 IU/ml than in patients with RF levels >204 IU/ml (≤204 IU/ml: 52.1; >204 IU/ml: 5.5), indicating that in patients with high RF levels, there was greater clinical benefit of CZP compared with ADA than in patients with low RF levels. We found that DAS28-CRP and DAS28-ESR were similar in CZP- and ADA-treated patients with high and low levels of ACPA. The absence of an impact of high levels of ACPA on response to CZP and ADA indicates that our observations were not confounded by ACPA levels as an autoantibody characteristic of RA that does not bind to the Fc-portion of IgG, hence suggesting a true biologic effect of RF.

We also found similar efficacy results when patients with high concentrations of anti-drug antibodies were excluded from the analysis. This suggests that anti-drug antibodies do not contribute to the difference observed in clinical outcomes between patients with RA and high RF levels when treated with either CZP or ADA.

These findings are consistent with previous reports from indirect comparisons that suggested consistent efficacy of CZP irrespective of baseline RF level, while patients with high RF levels who received Fc-containing TNFis appeared to have lower drug concentrations and response to therapy [20, 25, 27, 28]. Of note, data from the current analysis have been derived from a randomized controlled trial which directly compared CZP with ADA.

Limitations of this analysis include that it was limited to the inclusion of only one Fc-containing TNFi; ETA, GOL and IFX were not assessed alongside CZP. Further, given that this is a *post hoc* analysis, the original study was not designed specifically to investigate outcomes in this subpopulation and therefore the study size and design was not sufficiently powered to find any differences between patients with high and lower RF levels. However, the head-to-head design of the *post hoc* EXXELERATE study supports the differentiation of CZP from Fc-containing TNFis in patients with RA and high RF levels in terms of drug concentrations and clinical responses. At the time of the EXXELERATE study, the relationship between RF levels and the efficacy of biologics in RA was not a widely considered concept. Therefore, it is unlikely that awareness of a patient's RF level would have confounded the outcomes reported in this post-hoc analysis. Finally, while smoking status at baseline data were not available, a real-world evidence study by Szekanecz *et al.* [34] found no effect of cigarette smoking on the response to CZP in Hungarian, Czech and Slovak patients with RA.

To conclude, patients with RA and high levels of RF are a subgroup of patients with poor clinical outcomes. In ADA-treated patients, clinical outcomes were poorer and drug concentration lower in patients with high RF levels compared with lower RF levels. However, in CZP-treated patients, those with high levels of RF had similar drug concentration and clinical outcomes to patients with lower levels of RF. Thus, the current data are the first from a head-to-head trial comparing CZP and ADA to show that response to therapy in patients with RA and high levels of RF is influenced by the presence or absence of an Fc-portion, which is in line with previous findings [27, 28].

## Supplementary Material

keae435_Supplementary_Data

## Data Availability

Underlying data from this manuscript may be requested by qualified researchers 6 months after product approval in the USA and/or Europe, or global development is discontinued, and 18 months after trial completion. Investigators may request access to anonymized individual patient-level data and redacted trial documents, which may include analysis-ready datasets, study protocol, annotated case report forms, statistical analysis plan, dataset specifications and clinical study reports. Prior to use of the data, proposals need to be approved by an independent review panel at www.Vivli.org and a signed data sharing agreement will need to be executed. All documents are available in English only, for a pre-specified time, typically 12 months, on a password protected portal.
