# Supplementary material for: Impact of high rheumatoid factor levels on treatment outcomes with certolizumab pegol and adalimumab in patients with rheumatoid arthritis
Source: Rheumatology (Oxford). 2024 Sep 2;63(11):3015–24. doi: 10.1093/rheumatology/keae435 (PMC11534142; doi:10.1093/rheumatology/keae435)
Supplement: keae435_Supplementary_Data [file keae435_supplementary_data.docx]

SUPPLEMENTARY MATERIAL


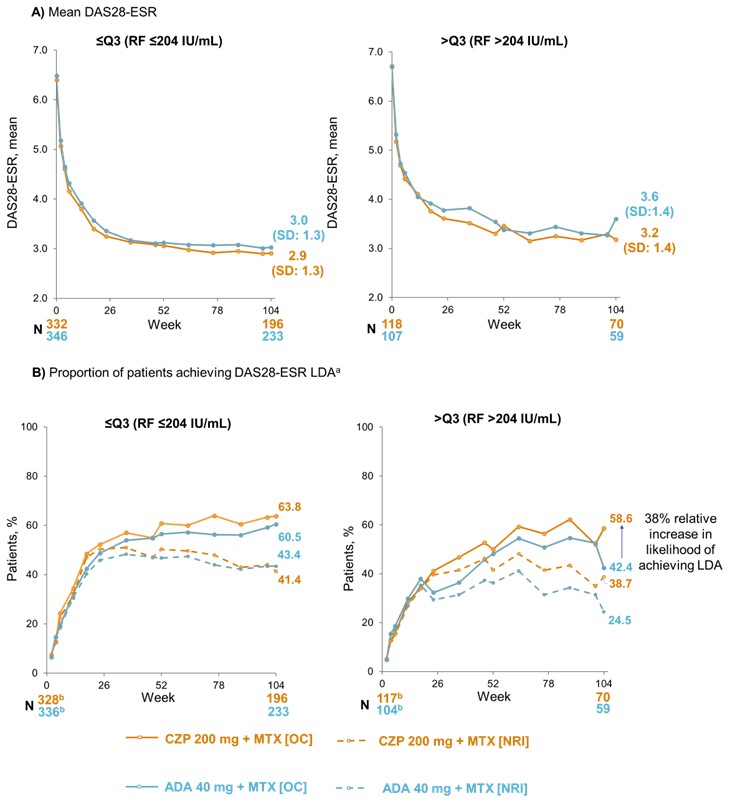
**Supplementary Figure S1.** Response to CZP and ADA to Week 104 measured by (A) DAS28‑ESR and (B) DAS28-ESR LDA, stratified by RF quartile at baseline [OC; NRI]

Full Analysis Set to Week 12; Week 12 Full Analysis Set from Week 18. Data reported according to the treatment patients were on at time of measurement (i.e., any patients who had switched TNFi at Week 12 were subsequently included in the arm for their new treatment, rather than the arm they were initially randomised to). N are for OC data. [a] Defined as DAS28-ESR ≤3.2. [b] N at Week 2. ADA: adalimumab; CZP: certolizumab pegol; DAS28: Disease Activity Score-28 joint count; ESR: erythrocyte sedimentation rate; LDA: low disease activity; MTX: methotrexate; NRI: non-responder imputation; OC: observed case; Q3: third quartile; RF: rheumatoid factor; SD: standard deviation; TNFi: tumour necrosis factor inhibitor.**Supplementary Figure S2.** Response to CZP and ADA to Week 104 measured by (A) SDAI and (B) SDAI LDA, stratified by RF quartile at baseline
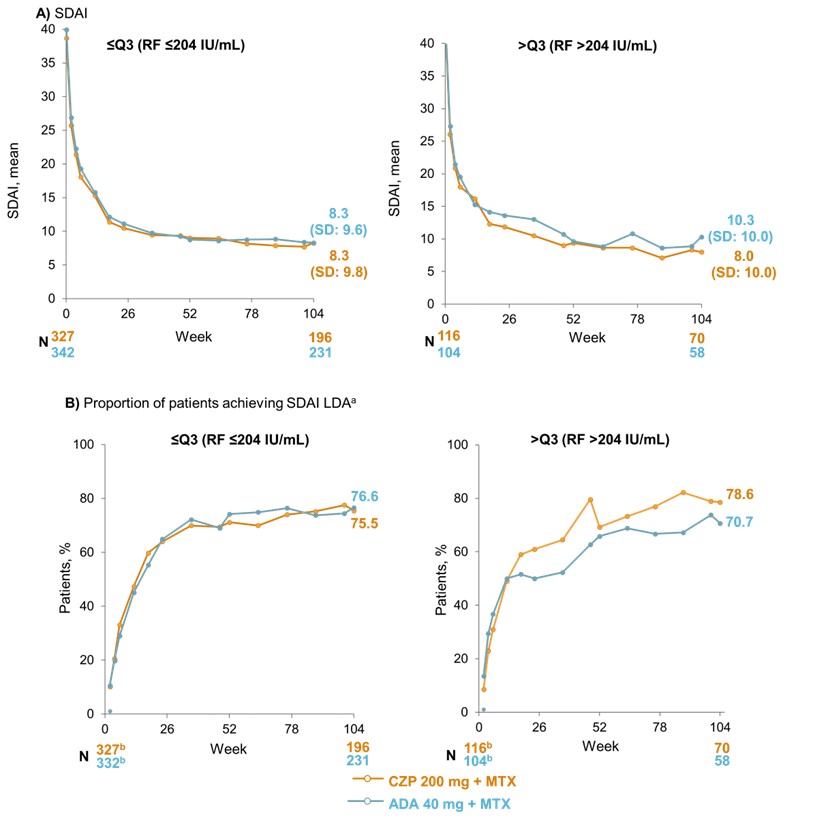
[OC]

Full Analysis Set to Week 12; Week 12 Full Analysis Set from Week 18. Data reported according to the treatment patients were on at time of measurement (i.e., any patients who had switched TNFi at Week 12 were subsequently included in the arm for their new treatment, rather than the arm they were initially randomised to). A high SDAI score indicates increased disease severity. [a] Defined as SDAI ≤11]. [b] N at Week 2. ADA: adalimumab; CDAI: Clinical Disease Activity Index; CZP: certolizumab pegol; MTX: methotrexate: OC: observed case; Q3: third quartile; RF: rheumatoid factor; SD: standard deviation; SDAI: Simple Disease Activity Index; TNFi: tumour necrosis factor inhibitor.


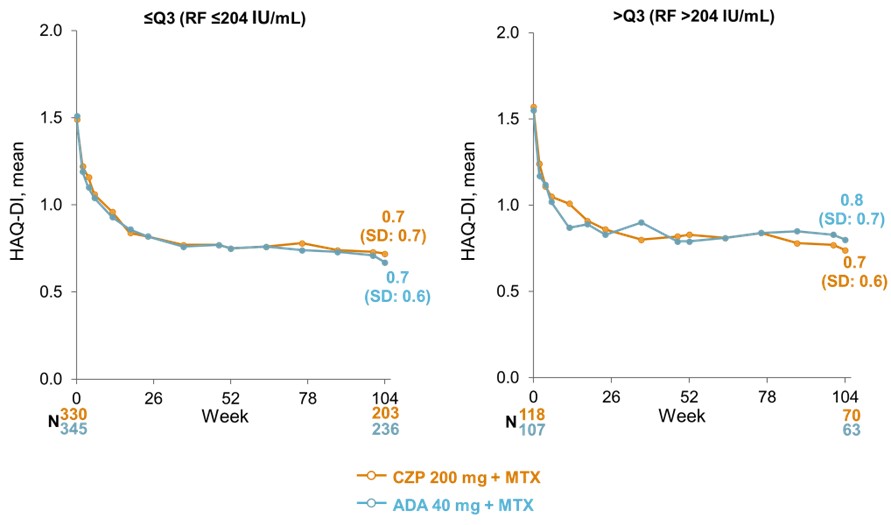
**Supplementary Figure S3.** HAQ-DI score in CZP and ADA treated patients tot Week 104, stratified by RF quartile at baseline [OC]

Full Analysis Set to Week 12; Week 12 Full Analysis Set from Week 18. Data reported according to the treatment patients were on at time of measurement (i.e., any patients who had switched TNFi at Week 12 were subsequently included in the arm for their new treatment, rather than the arm they were initially randomised to). ADA: adalimumab; CZP: certolizumab pegol; MTX: methotrexate: HAQ-DI: Health Assessment Questionnaire-Disability Index; OC: observed case; Q3: third quartile; RF: rheumatoid factor; SD: standard deviation; TNFi: tumour necrosis factor inhibitor.**Supplementary Figure S4.** Response to CZP and ADA to Week 104 measured by the proportion of patients achieving Boolean remission, stratified
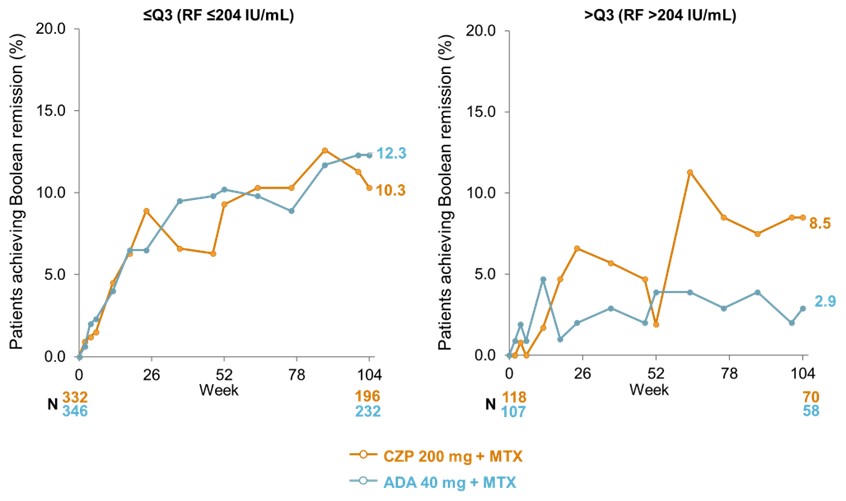
by RF quartile at baseline [NRI]

Full Analysis Set to Week 12; Week 12 Full Analysis Set from Week 18. Data reported according to the treatment patients were on at time of measurement (i.e., any patients who had switched TNFi at Week 12 were subsequently included in the arm for their new treatment, rather than the arm they were initially randomised to). Boolean remission is defined as SJC≤1 and TJC≤1 and PtGADA≤2 and CRP≤1. ADA: adalimumab; CRP: C-reactive protein; CZP: certolizumab pegol; MTX: methotrexate; NRI: non-responder imputation; PtGADA: Patient’s Global Assessment of Disease Activity; Q3: third quartile; RF: rheumatoid factor; SJC: swollen joint count; TJC: tender joint count; TNFi: tumour necrosis factor inhibitor.

**Supplementary Figure S5.** Mean DAS28-CRP and DAS28-ESR in CZP and ADA treated patients after exclusion of patients with high levels of anti-drug antibodies (>50^th^ percentile), stratified by RF quartile, to Week 104 [OC]
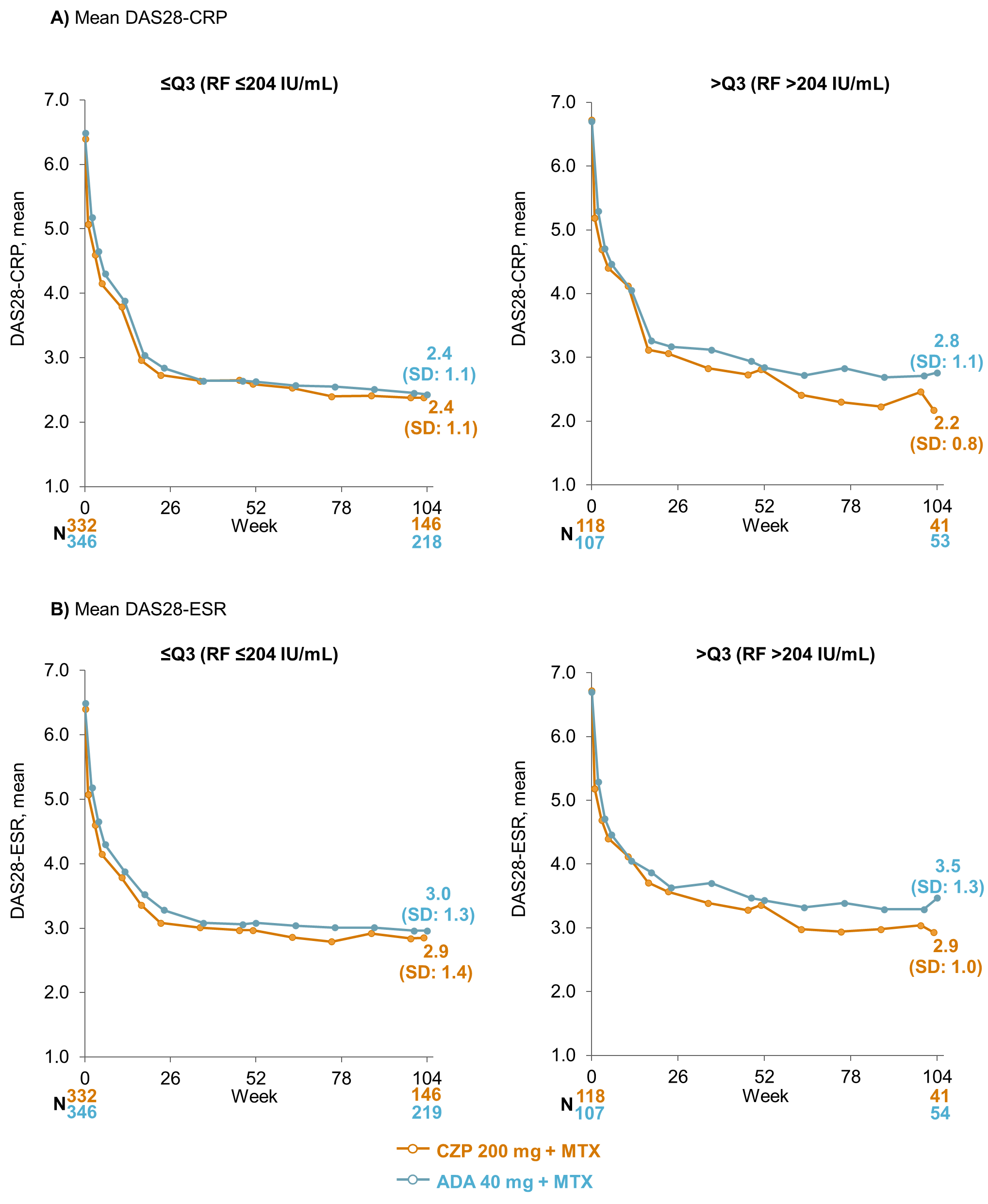


Full Analysis Set to Week 12; Week 12 Full Analysis Set from Week 18. Data reported according to the treatment patients were on at time of measurement (i.e., any patients who had switched TNFi at Week 12 were subsequently included in the arm for their new treatment, rather than the arm they were initially randomised to). ADA: adalimumab; CRP: C-reactive protein; CZP: certolizumab pegol; DAS28: Disease Activity Score-28 joint count; ESR: erythrocyte sedimentation rate; MTX: methotrexate: OC: observed case; Q3: third quartile; RF: rheumatoid factor; SD: standard deviation; TNFi: tumour necrosis factor inhibitor.


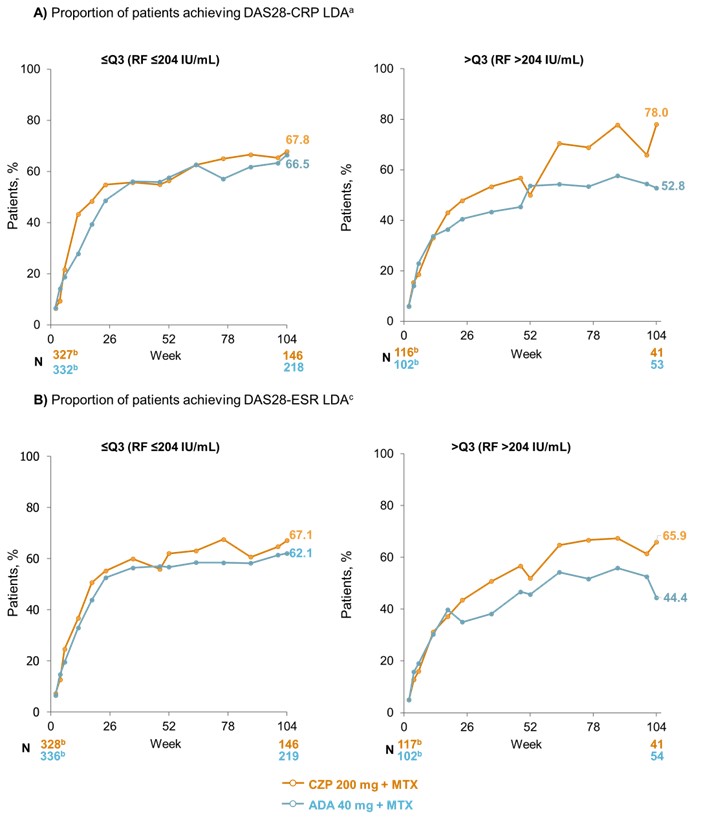
**Supplementary Figure S6.** Mean DAS28-CRP LDA and DAS28-ESR LDA in CZP and ADA treated patients after exclusion of patients with high levels of anti-drug antibodies (>50^th^ percentile), stratified by RF quartile, to Week 104 [OC]

Quartiles indicate RF level at baseline. Full analysis set. Data reported according to the treatment patients were on at time of measurement (i.e., any patients who had switched TNFi at Week 12 were subsequently included in the arm for their new treatment, rather than the arm they were initially randomised to). [a] Defined as DAS28-CRP ≤2.7. [b] N at Week 2. [c] Defined as DAS28-ESR ≤3.2. ADA: adalimumab; CRP: c-reactive protein; CZP: certolizumab pegol; DAS28: Disease Activity Score-28 joint count; ESR: erythrocyte sedimentation rate; LDA: low disease activity; MTX: methotrexate; OC: observed case; Q3: third quartile; RF: rheumatoid factor; TNFi: tumour necrosis factor inhibitor.
